# Molecular Basis of Live-Attenuated Influenza Virus

**DOI:** 10.1371/journal.pone.0060413

**Published:** 2013-03-26

**Authors:** Wen He, Wei Wang, Huamin Han, Lei Wang, Ge Zhang, Bin Gao

**Affiliations:** 1 CAS Key Laboratory of Pathogenic Microbiology and Immunology, Institute of Microbiology, Chinese Academy of Sciences, Beijing, PR China; 2 Hebei Key Laboratory of Medical Biotechnology, Shijiazhuang, PR China; 3 Graduate University of Chinese Academy of Sciences, Beijing, PR China; 4 China-Japan Joint Laboratory of Molecular Immunology and Microbiology, Institute of Microbiology, Chinese Academy of Sciences, Beijing, PR China; 5 School of Life Sciences, University of Science and Technology of China, Hefei, China; Instituto Butantan, Brazil

## Abstract

Human influenza is a seasonal disease associated with significant morbidity and mortality. The most effective means for controlling infection and thereby reducing morbidity and mortality is vaccination with a three inactivated influenza virus strains mixture, or by intranasal administration of a group of three different live attenuated influenza vaccine strains. Comparing to the inactivated vaccine, the attenuated live viruses allow better elicitation of a long-lasting and broader immune (humoral and cellular) response that represents a naturally occurring transient infection. The cold-adapted (*ca*) influenza A/AA/6/60 (H2N2) (AA *ca*) virus is the backbone for the live attenuated trivalent seasonal influenza vaccine licensed in the United States. Similarly, the influenza A components of live-attenuated vaccines used in Russia have been prepared as reassortants of the cold-adapted (*ca*) H2N2 viruses, A/Leningrad/134/17/57-*ca* (Len/17) and A/Leningrad/134/47/57-*ca* (Len/47) along with virulent epidemic strains. However, the mechanism of temperature-sensitive attenuation is largely elusive. To understand how modification at genetic level of influenza virus would result in attenuation of human influenza virus A/PR/8/34 (H1N1,A/PR8), we investigated the involvement of key mutations in the PB1 and/or PB2 genes in attenuation of influenza virus *in vitro* and *in vivo*. We have demonstrated that a few of residues in PB1 and PB2 are critical for the phenotypes of live attenuated, temperature sensitive influenza viruses by minigenome assay and real-time PCR. The information of these mutation loci could be used for elucidation of mechanism of temperature-sensitive attenuation and as a new strategy for influenza vaccine development.

## Introduction

Influenza A viruses belong to *Orthomyxoviridae* family viruses and are highly contagious pathogens for both human and animals. As a major cause for winter respiratory infection, seasonal influenza contributes the biggest number of morbidity and mortality each year. Annual epidemics results in about three to five million cases of severe illness and about 250,000–500,000 deaths worldwide [1].

Vaccination is the primary strategy for the prevention and control of influenza. Two different types of vaccines, inactivated and live attenuated viruses are currently licensed for the prevention of seasonal influenza [2], [3], [4], [5]. The trivalent inactivated influenza virus vaccine (TIV) has been used since 1945. Each dose is formulated to contain three viruses (or their HA proteins) representing the influenza A H3N2, influenza A H1N1, and influenza B strains chosen to be the most likely strains to circulate in the upcoming influenza season [6]. Three components are updated annually as needed on the basis of national and international recommendations [7], [8], [9].

Reassortants of cold-adapted (*ca*) influenza donor strains have been used as live -attenuated vaccines for direct administration to the respiratory tract. In USA, influenza A vaccines have been derived from a cold-adapted influenza donor strain originally prepared by serial passage of the H2N2 virus Ann Arbor/6/60 (A/AA/6/60) 32 times in chicken kidney cultures at successively lower temperatures to 25°C to produce a cold-adapted donor strain A/AA/6/60-*ca* [10]. The AA *ca* virus displays important phenotypes that have been crucial to the development of the virus for clinical use. The virus replicates efficiently at 25°C and 33°C but does not replicate well at temperatures above 39°C; these phenotypic traits have respectively been designated cold-adapted (*ca*) and temperature-sensitive (*ts*) phenotypes. In addition, the AA *ca* virus is attenuated *in vivo* (*att* phenotype) and is restricted in replication in the respiratory tract of mice and ferrets [9]. These phenotypes are specified by five mutations in three gene segments (PB1, PB2, and NP) [11].

The influenza A viral genome is composed of eight negative sense single-stranded RNA segments (vRNAs) that together encode 10–11 proteins. Live attenuated influenza vaccines (LAIVs) are 6:2 genetic reassortants that are currently produced using reverse genetics, in which the six internal protein gene segments (PB2, PB1, PA, NP, M and NS) are derived from the vaccine donor strains (A/Ann Arbor/6/60 H2N2 or B/Ann Arbor/1/66), reassorted with the hemagglutinin (HA) and the neuraminidase (NA) gene segments from the appropriate contemporary wild-type (wt) viruses [12].

In Russia two *ca* influenza A donor strains have been prepared by a similar approach with some modifications. The two donor strains, each derived from the same parental H2N2 strain A/Leningrad/134/57 (Len/57) [13], which had been adapted to grow in embryonated chicken eggs at 25°C. The first donor strain, Len/17, was obtained after a total of 17 passages of Len/57 and has been used in the preparation of vaccines for use in adults. The second, Len/47, was passaged a total of 47 times and has been used in vaccines for children less than 16 years old [14].

The development of live, attenuated, cold-adapted, trivalent influenza vaccine given intranasally may improve vaccine usage and provide a simple and convenient method for the prevention of influenza. LAIVs are well suited to prevent pandemic and epidemic influenza and may provide distinct advantages over inactivated or subunit vaccines. Data from three large placebo-controlled clinical studies indicate that relatively high levels of efficacy (ranging from 60% to 90%) were seen in previously unvaccinated young children after a single dose of trivalent LAIV [15]. Techniques to update the antigenic composition of live, attenuated vaccine annually have shown that new antigens can be reliably conferred in the trivalent live vaccine and that the live vaccine has predictable levels of attenuation, immunogenicity, and efficiency.

The route of vaccination is important in determining the immune response, as the initial site of pathogen invasion influences the efficacy of protection [16]. This vaccine is delivered through intranasal administration and allows elicitation of a long-lasting, broader immune (humoral and cellular) response, which more closely resembles natural immunity [17]. On the other hand, several trials have reported that LAIVs can boost virus-specific cytotoxic T lymphocytes (CTLs) as well as mucosal and serum antibodies and provide broad cross-protection against heterologous human influenza A viruses, including the avian virus [18], [19], [20], [21], [22]. Live vaccines increase the production of CTLs and antibodies that protect the upper respiratory tract, and may protect against infection [23], [24]. The integrated approach provided evidence for the induction of cross-protective immunity by primary infection to hetero-subtypic influenza A strains [25].

Although live attenuated influenza vaccines have been approved for clinical use, their mechanisms have not been completely understood. The viral RNA polymerases are highly conserved in strains of influenza virus infecting both birds and human beings [26]. Mutational analyses indicate that the C-terminus of PB1 (residues 712–746) forms the interaction core with the N-terminus of PB2 [27]. Immunoprecipitation assay and deletion mutation assay demonstrated that the N-terminal 249 amino acid residues of PB2 are responsible to bind to PB1 [28]. To elucidate the mechanism of attenuation of LAIV, the PB2 and PB1 genes of A/PR8 (H1N1) were mutated by means of reverse genetics and the temperature sensitive, attenuated viruses were identified. We have analyzed the activities of polymerases and RNA expression levels in these temperature sensitive viruses. Our studies suggested that these mutation loci for the preparation of LAIV could be useful for vaccine design against seasonal influenza.

## Results

### 1, Identification of key residues involved in *ts* phenotypes of A/PR8

**Figure 1:**
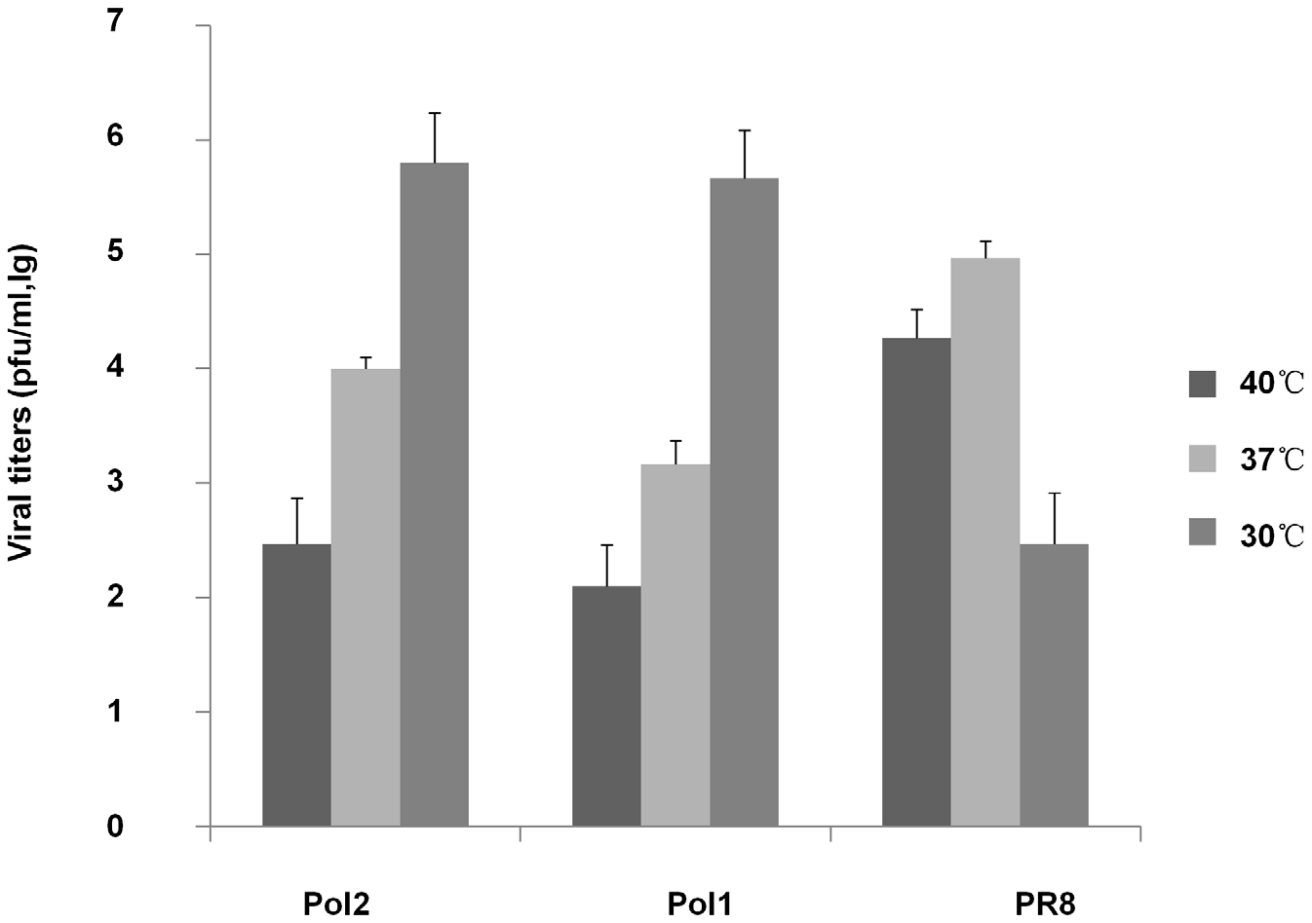
Comparison of different virus titers produced in infected MDCK cells at different temperatures. MDCK cells were infected with viruses (1×10^4^ pfu ) at 40°C, 37°C and 30°C individually. Supernatants were collected after 2 days. The yield of progeny viruses in MDCK supernatants was determined by plaque titration assay. P<0.05

To find the key amino acids responsible for expression of the *ts* and *att* phenotype, PB1 and PB2 genes of A/PR8 were modified by site-directed mutagenesis according to sequence information of AA-*ca* and Len/17 (Table 1 and Table 2). Many different mutant viruses were made with different combination of key residues. Two of these mutants: Pol1 with one mutation site in PB2 (PB2-478) and two mutation sites in PB1 (PB1-265-591); and Pol2 with two mutation sites in PB1 (PB1-391-581) demonstrated typical *ts* phenotype. As shown in Figure 1, the titers of Pol1 or Pol2, carrying three or two mutant loci derived from Len/17 or AA-*ca*, were reduced by 10^3^ pfu/ml at 40°C, comparing to that at 30°C. By contrast, the wt A/PR8 was not able to grow effectively at 30°C and its virus titer was much higher than ones of Pol1 or Pol2 at 40°C.

**Table 1:** Mutation loci from Ann-ca.

Ts site	Wt A/PR8	Ann-ca	Ts phenotype	Att phenotype
PB1 : 391	K	E	+	+
581	E	G	+	+
661	A	T	--	--
PB2:265	N	S	--	--

**Table 2:** Mutation loci from Len-ca.

Ts site	Wt A/PR8	Len-ca	Ts phenotype	Att phenotype
PB1:265	K	N	+	+
591	V	I	+	+
PB2:478	V	L	+	+
PA:28	L	P	--	--
341	V	L	--	--

### 2, The key residues identified play an important role in influenza viral RNA synthesis

**Figure 2:**
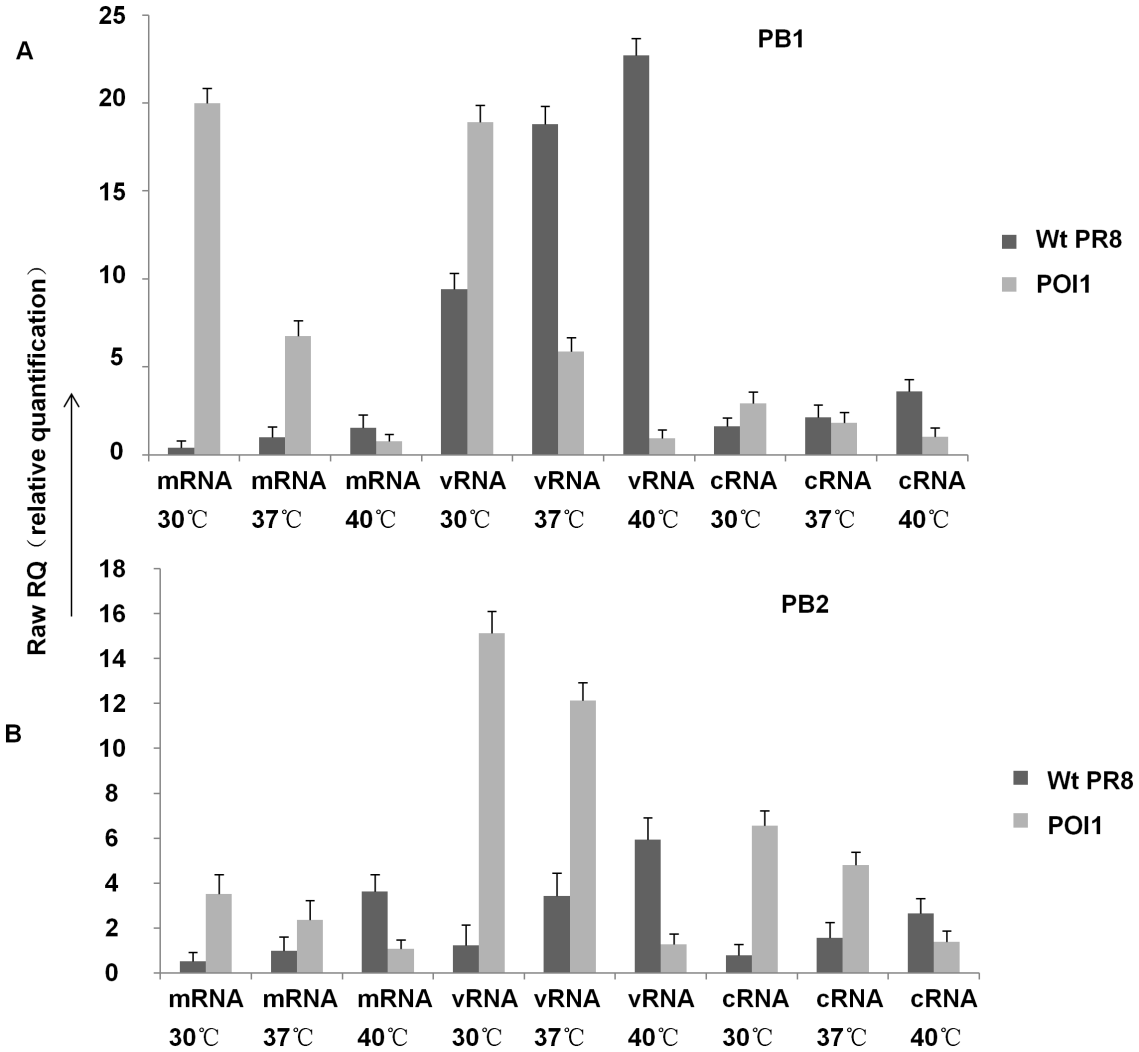
Mutations affect viral RNA synthesis at different temperatures. Viral RNAs in MDCK cells infected with the different viruses (1×10^4^ pfu ) were analyzed by real-time PCR. Cells were harvested at 16h p.i. (A) mRNA, cRNA, vRNA levels of PB1 from MDCK infected with A/PR8 & Pol1; (B) mRNA, cRNA, vRNA levels of PB2 from MDCK cells infected with A/PR8 & Pol1. P<0.05.

To detect whether the key residues affect viral RNA synthesis, we examined viral RNA synthesis in cells infected with different viruses at 30 °C, 37 °C, 40 °C. The viral PB1, PB2, NP RNA level was monitored at 16 hpi (Figure 2) in MDCK cells infected with 1×10^4^ pfu of viruses. The Pol1-infected cells showed decreased PB1, PB2, NP RNA synthesis following the increased temperature contrary to that in wt virus. The real-time quantitative PCR assays were carried out with a primer set specific for PB1, PB2, NP RNA, showing that RNA synthesis is severely curtailed by these mutations.

### 3, Genetic modifications in the PB2 and PB1 genes of A/PR8 strain result in impaired polymerase activity

**Figure 3:**
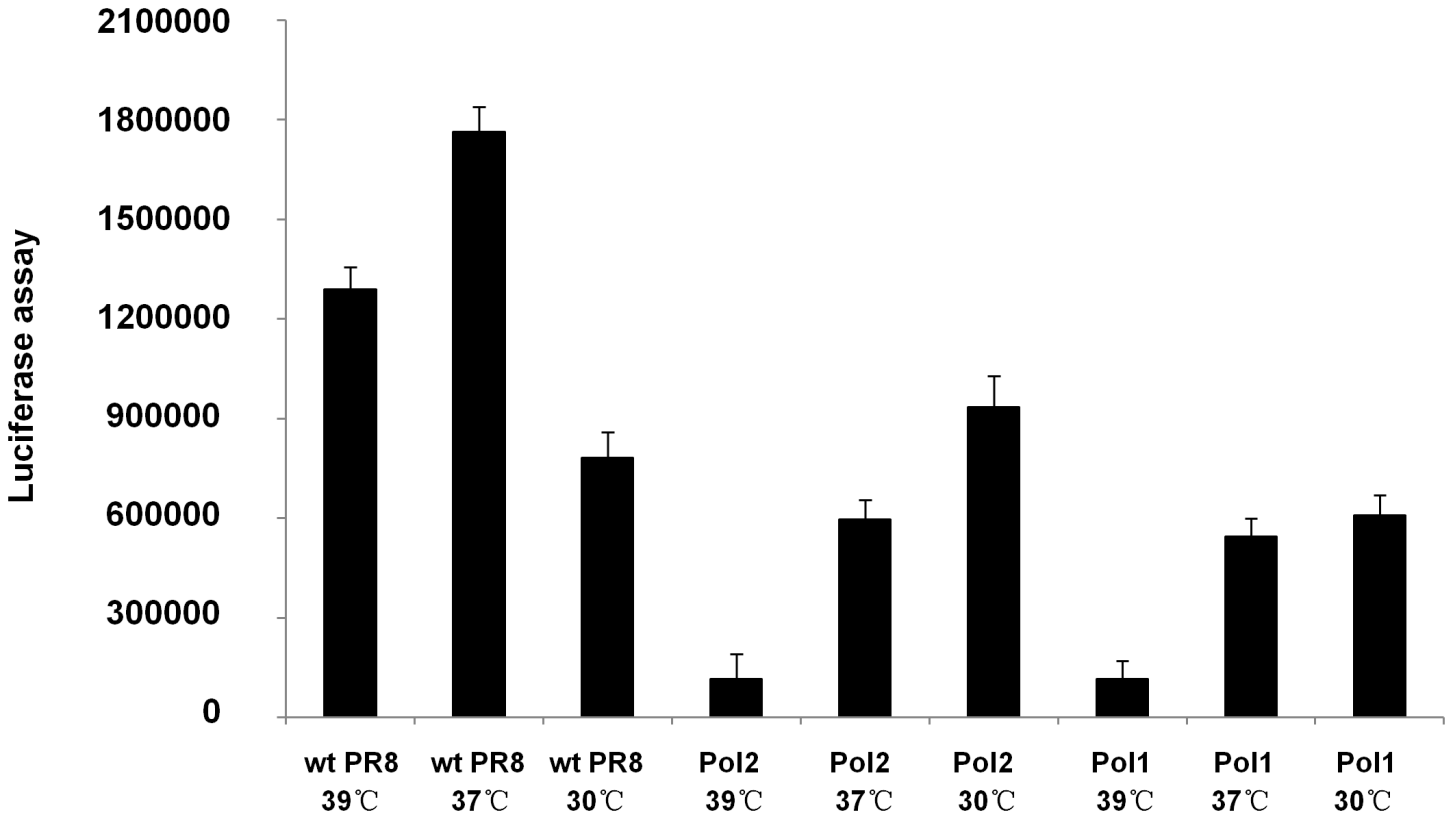
Mutations in PB1 and PB2 influenced polymerase activities of A/PR8 virus strain. Mutant and wt polymerases activities at different temperatures were analyzed by minigenome assays. The luciferase expression was quantified as described in Materials and Methods. Mock-transfected cells were also analyzed as negative control. Cells were lysed after 48 h. The result was assayed by the luminometer. P<0.05

To evaluate whether the modification introduced into the PB2 and PB1 genes influences polymerase activity, we performed a flu minigenome reporter assay as described in Materials and Methods. The results at 36−48 hours after transfection showed that the optimal temperature for wt polymerase transcriptional activity was 37°C. In contrast, polymerase activities in virus mutants (Pol1 and Pol2) were greatly impaired at 37°C. This reduced polymerase activity could be recovered for the *ts* polymerases at 30°C (Figure 3). More importantly, the *ts* polymerase activities at 39°C were significantly reduced, which confirms the restrictive temperature phenotype of the polymerase mutations in A/PR8 virus.

### 4, Viruses that carry the mutated polymerase complex are attenuated *in vivo*

**Figure 4:**
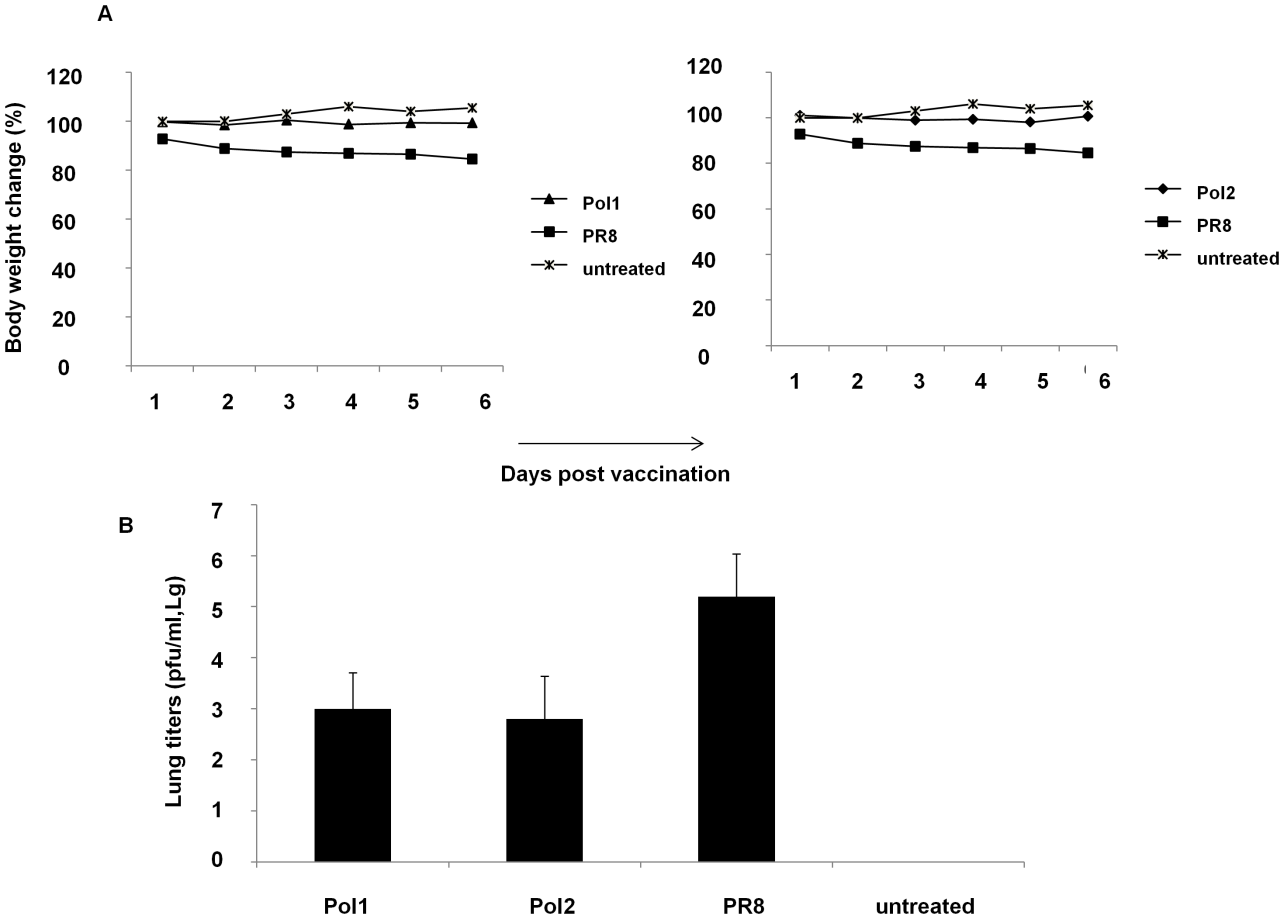
Mutated viruses are less toxic to mice than wild type of A/PR8. A. Body weight changes of C57/B6 mice inoculated with Pol1, Pol2, A/PR8 or mock infected. Body weight was monitored daily. B. Lungs’ viral titers in C57/B5 mice inoculated with different viruses. Five mice in each group were sacrificed on day 3 and 6 post vaccination. Lungs were homogenized in PBS to make 10% (wt/vol) (lungs) tissue homogenates, and viral titers were determined by TCID_50_ assay. Data were analyzed on day 6 after vaccination. P<0.05

In order to determine whether the *ts* mutations would provide an *att* phenotype *in vivo,* we tested two A/PR8 mutants in a mouse model of influenza infection. A/PR8 has been previously shown to confer a virulent phenotype in mice [29]. To test whether viruses with mutations are attenuated *in vivo,* several groups of mice were inoculated with 10^7^ pfu of wt viruses along with mutants. A mock group was inoculated with PBS. After inoculation, the weight of mice was monitored daily as indication of toxicity for 10 days. As shown in Figure 4A, the body weight of mice inoculated with the A/PR8-Pol1 or A/PR8-Pol2 virus did not change during the observation period the same as that in mock-infected mice.

Accordingly, the amount of viruses presented in the mice lungs are lower in animals infected with mutant viruses than that in mice infected with wt A/PR8 (Figure 4B). The level of replication of A/PR8-Pol1, A/PR8-Pol2 and A/PR8 wt viruses was compared in the respiratory tract of mice. The mean virus titer over day 3, and 6 in the lungs of mice inoculated with the A/PR8 wt virus was 10^5.2^ pfu/ml. By contrast, the titer of virus from lungs in mice inoculated with A/PR8-Pol1 or A/PR8-Pol2 was 10^3^ pfu/ml, 10^2.8^ pfu/ml respectively. Thus, the A/PR8 *att* virus was restricted in the respiratory tract of mice compared to the A/PR8 wt virus (P<0.05).

### 5, Attenuated influenza viruses induced immune responses

**Figure 5:**
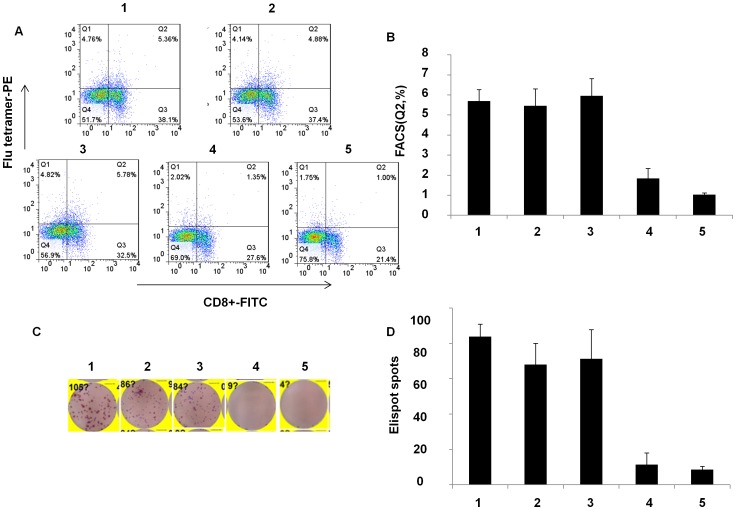
Analysis of virus-specific CTL by tetramer-staining and Enzyme-linked immunospot (ELISPOT) for IFN-γ. A, B. Tetramer-staining by CD8+−FITC and flu-tetramer-PE. The splenocytes were incubated with CD8+−FITC and flu-tetramer-PE. The stained cells were analyzed by flow cytometry with a Guava Easycyte. All FACS data were analyzed using FlowJo software v7.2.5. C,D. Elispot assay was done according to Mouse IFN- γ Elisport kit. Spots were counted by an Elispot reader (Cellular technology Ltd). P<0.05 1. Spleen cells (C57 immunized by A/PR8 intranasal, 1×10^4^ pfu); 2. Spleen cells (C57 immunized by Pol2 intranasal, 1×10^4^ pfu); 3.spleen cells (C57 immunized by Pol1 intranasal, 1×10^4^ pfu); 4. Spleen cells (C57 immunized by inactivated A/PR8); 5. Spleen cells (C57 immunized by PBS)

**Figure 6:**
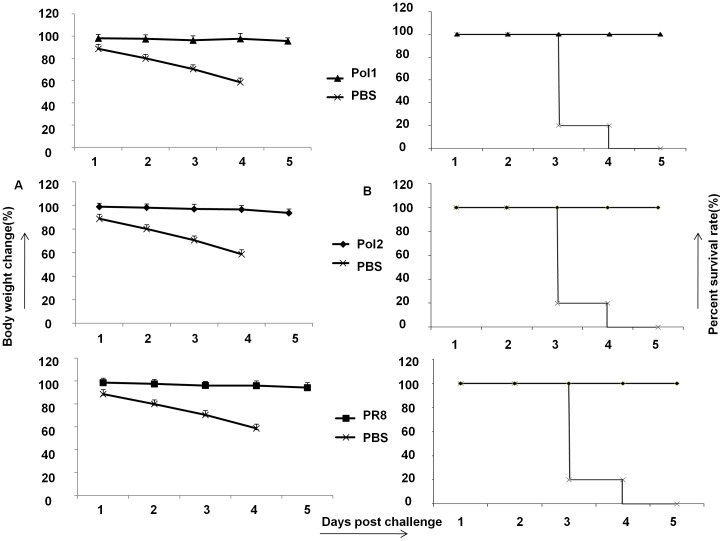
Protection against flu virus challenge in mice immunized by A/PR8 mutants. A. Body weight loss in vaccinated/challenged C57/B6 mice. Mice were vaccinated with Pol1, Pol2, A/PR8 or mock vaccinated and challenged intraperitoneally on day 21 post-immunization with a lethal dose of A/PR8 (1×10^7^ pfu ). Body weight was monitored daily. B. Survival rate of mice immunized by Pol1, Pol2, A/PR8 against lethal dosage challenge.

As a live virus, we expected that the attenuated mutants would produce not only protective antibodies but also strong T cell immune response. Since the level of HAI antibodies closely correlates to an influenza vaccine protection, we analyzed anti- hemagglutinin antibodies in mice inoculated with attenuated influenza viruses. HAI antibodies were stimulated by mutant strains as strong as that produced by wt A/PR8. The advantage of LAIVs is that the immune response includes not only antibody production but also T cell stimulation. To demonstrate T cell response, splenocytes of mice were collected on day 6 after immunization with A/PR8, NP_366–374_ specific T lymphocytes in spleens derived from mice immunized by A/PR8-Pol1 and A/PR8-Pol2 were analyzed by tetramer staining. As shown in Figure 5A and 5B, specific T cells up to 5.69%, 5.96% of total splenocytes were readily detected in mice inoculated with A/PR8-Pol1, or A/PR8-Pol2 respectively, comparable to a specific 5.46% CD8+T lymphocyte population in the spleen of mice immunized with A/PR8 viruses. As expected, the specific tetramer staining cells were virtually absent in mouse group injected with PBS as control. Similarly, Elispot data were consistent with FACS results (Figure 5C, D). Finally, the protection of immunized mice with attenuated viruses against the challenge of lethal dose of wt viruses was demonstrated. In these experiments, a group of mice were inoculated intranasally with 10^4^ pfu of A/PR8-Pol1, A/PR8-Pol2 and both of WT and mock as control. HAI assays demonstrated that A/PR8 mutants elicited neutralizing antibodies in all of immunized mice, with the average antibody titer of each group ranging from 2^−10^ to 2^−11^. When the mice were challenged with lethal dose of A/PR8, each of groups immunized by A/PR8 mutants or prior infection by the wt virus protected the mice. No disease symptoms were observed in the mice immunized by A/PR8 viruses or its mutants. In contrast, non-immunized mice developed the typical symptoms including ruffled fur, hunched posture, and weight loss as early as 1 day post challenge and the animals showed statistically significant (p < 0.05, Student’s t-test) weight loss compared to the wt or mutated A/PR8 immunized mice (Figure 6). The symptoms progressed to severe disease, and the mice became moribund, and succumbed to infection by 3–5 dpc.

## Discussion

The influenza polymerase is essential to the biological processes of (i) virus replication in cells by replicating the vRNA segments and transcribing their genes and (ii) virus evolution through its error-prone RNA replication, leading to viruses that are better adapted to new host species [16].

The three largest vRNAs encode the three subunits of the RNA-dependent RNA polymerase: the acidic subunit PA and the two basic subunits PB1 and PB2. PB1 is the core subunit of the viral RNA-dependent RNA polymerase, which possess RNA polymerase catalytic activity. PB2 is responsible for the binding, cleavage and recruitment of cellular mRNA cap-1 structures essential for viral mRNA synthesis [17], [18]. Mutational analysis indicate that the C-terminus of PB1 (residues 712–746) forms the core interaction with the N-terminus of PB2 [30].

The mutations responsible for the *ts* phenotype in the A/Ann Arbor/6/60 *ca* influenza virus strain have been mapped to the following 5 amino acids in the viral polymerases: N265S in PB2, K391E, E581G, and A661T in PB1, and D34G in NP [11]. A previous report also demonstrates that the sequential introduction of the *ts* mutations identified in other influenza virus strains into the PB2 gene of the A/AA/6/60 reassortant results in increased *ts* and *att* phenotypes [19]. A.I. Klimov *et al.* suggest that mutations in the PB2 (V-478-L) and PB1 (K-265-N, V-591-I) genes play a critical role in the attenuation of the *ca* Len/17 vaccine donor strain [**31**] **.**

In this study we demonstrated that the K391E and E581G amino acid changes in the PB1 gene independently contributed to attenuation of A/PR8. Although related reports have been described [11], we thought the K391E and E581G amino acid changed in the PB1 gene are two key mutations and other mutations’effects can not be observed. Additionally, the K265N and V591I amino acid changed in the PB1 gene and V478L amino acid changed in the PB2 gene have also been proved for the first time to contribute to the attenuation of A/PR8. Just two or three mutations for the preparation of LAIV could be useful for influenza pandemic preparedness and seasonal influenza. The availability of alternative backbones to A/PR8 strain should be considered for influenza vaccine development. The limited replication of the att viruses should make them safe vaccines for human use. Having different alternative LAIV backbones available should aid in our strategy for the development of better cross-protective vaccines against influenza.

The *ts* and *att* phenotypes can be produced by site-directed mutagenesis of polymerase genes [21]. To evaluate if the modifications introduced in the PB2 and PB1 genes produced a *ts* phenotype, we performed a flu minigenome reporter assay to analyze viral polymerase function. Using this system, we confirmed that the specific changes in both the PB1 and PB2 gene segments are responsible for *ts* and *att* phenotype of A/PR8.

Results of this study demonstrated that mutations in polymerase genes were essential for attenuation of A/PR8. They independently contributed to attenuation of the A/PR8 strain. Importantly, mutations in both these genes were responsible for the manifestation of the *ts* phenotype, but they didn’t lead to the *ca* phenotype.

The mutation loci harboring the A/PR8- PB1 or PB2 segment exhibited a reduced ability to replicate in the lungs of mice and decreased activity of polymerase, indicating that these segments play a role in expression of the *att* phenotype. The recombinant viruses in which the PB1 and PB2 mutation loci were derived from *ca* A/AA/6/60 and A/Leningrad/134/17/57. This study suggests that two gene segments, PB1, PB2, confer the *att* phenotype in the mice model. These two gene segments also include the loci responsible for exhibition of the *ts* phenotype. Transferring the *ts* loci into a divergent A/PR8 strain also resulted in transferring the *ts* and *att* phenotypes to these recombinants. The modified A/PR8 strain with the *ts* and *att* phenotypes become non-virulent for mice.

Mice immunized with the mutated strains were asymptomatic, and the lung tissue titers in mice infected with mutant viruses were lower than the lung tissue titers in mice infected with WT virus, indicating that they were significantly attenuated *in vivo*. Protective immunity following challenge infection was assessed by determining survival rates, weight loss, lung virus titers, and serum antibodies. These results were correlated with the detection of virus specific CTL responses before and after challenge infection by tetramerstaining and Elispot assay.

In summary, we proposed that key mutations of influenza virus could lead to temperature-sensitive attenuated phenotype, partly due to impaired activity of polymerase and decreased synthesis of viral RNA.

## Materials and Methods

### Ethics statement

All animals were cared for and maintained under the supervision and guidelines of the Institutional Ethic Committee of the Institute of Microbiology, Chinese Academy of Sciences (permit number CASPMI009,CASPMI011). All surgery was performed under sodium pentobarbital anesthesia, and all efforts were made to minimize suffering.

### Cell culture

Human embryonic kidney (293T) cells and Madin-Darby canine kidney (MDCK) cells from ATCC were maintained in Dulbecco’s modified Eagle’s medium (DMEM) supplemented with 10% fetal bovine serum (FBS). Human lung adenocarcinoma (A549) cells from cell bank of Chinese Academy of Medical Sciences were maintained in RPMI-1640 medium, supplemented with 10% FBS.

### The generation of A/PR8 vaccine seeds stock

The A/PR8 virus used in this study was generated using plasmid-based reverse genetics as previously described (14). 293T cells were maintained in DMEM containing 10% FBS. For the transfection, confluent 293T cells in a 10 cm^2^ culture plate were trypsinized on the day before transfection; 15 ml of this cell suspension was seeded into 10 cm^2^ plate and cultured for 24 hours. Calcuim phosphate transfection method was used. Briefly, the cell culture was replaced by Opiti-medium before the transfection; eight plasmids (kindly provided by Dr Kenth Gustafsson at UCL) per 4 µg of DNA were mixed, incubated at room temperature for 15 minutes, and added to the cells. Six hours later, the DNA-transfection mixture was replaced by DMEM containing 10% FBS. The cell supernatant was harvested 48 hours after transfection. Viruses were propagated in the allantoic cavities of 9 to 11-day-old embryonated chicken eggs (Beijing Merial Vital Laboratory Animal Technology Ltd). The eggs were incubated at 37°C for 48 hours and then were chilled at 4°C overnight. After the harvest of allantoic fluid, all eggs were autoclave sterilizated and then disposed as bioharzards according to the institutional specified rules for biohazardous substances. The virus titer of the allantoic fluid was determined by titration of the supernatant on MDCK cells.

### Generation of mutant viruses

Site-directed mutagenesis to introduce specific changes into the PB1, PB2, PA gene was performed using a QuikChange site-directed mutagenesis kit (Stratagene, La Jolla, CA), and the sequences were confirmed by sequence analysis. The candidate vaccine strains were generated by cotransfecting eight cDNA plasmids into cultured 293T cells. Viruses were propagated in the allantoic cavities of 9 to 11-day-old embryonated chicken eggs (Beijing Merial Vital Laboratory Animal Technology Co., Ltd). The virus titer of the allantoic fluid was determined by titration of the supernatant on MDCK cells.

### Comparison of virus titers between mutant and wt viruses at different temperatures

MDCK cells were infected with Pol1,Pol2, wt A/PR8 viruses (1×10^4^ pfu ) at 40°C(restrictive temperature), 37°C(permissive temperature for wt) and 30°C(permissive temperature for mutants) individually. Supernatants were collected after 2 days. The virus titer was determined by titration of the supernatant on MDCK cells.

### Transient transfection and minigenome assay

To test the transcription efficiency of the modified influenza virus polymerases at different temperatures, a minigenome assay was performed in 293T cells. Briefly, plasmids encoding wild-type or mutant PB2 and PB1 along with wt versions of PA and NP were co-transfected with the influenza virus replicon reporter plasmid pPOLI-luciferase [32]. Briefly, 0.5 µg of each plasmid was mixed, after 15 minutes of incubation at room temperature (RT), the mixture was added onto 80% confluent 293T cells seeded the day before in six-well plates. The transfections were set up at the following different temperatures: 30°C, 37°C, and 39°C. At 48 hours post-transfection, the cells were treated with cell lysis buffer, centrifuged, and supernatants were collected. Add 5 µl aliquots of cell lysate to individual luminometer tubes containing 180 µl of luciferase assay buffer at room temperature. To start the assay, inject 100 µl of luciferin solution into the luminometer tube and measure the light output in the luminometer.

### Replication kinetics in cell cultures

Confluent monolayers of MDCK cells were infected with A/PR8 or any recombinant viruses (1×10^4^ pfu) at 37°C in triplicate. One hour post infection (hpi), inoculation solution was removed, and cells were washed twice and DMEM supplemented with 1.4% BSA, 2.5 µg/ml TPCK-trypsin, and 0.1% antibiotic (Penicillin Streptomycin, invitrogen) was added. Supernatants were collected at 2, 12, 24, 48, and 72 hpi.

### Real-time PCR analysis

MDCK cells infected with influenza virus (1×10^4^ pfu) were grown to about 90% confluence. The inoculation medium was removed after 1 h, and cultured in DMEM supplemented with 1.4% BSA, 2.5 µg/ml TPCK-trypsin, and 0.1% antibiotic for 16 h. Cells were scraped off, washed twice with PBS, and collected by centrifugation (500 g for 5 min). Total RNA was prepared using the RNApure total RNA fast isolation kit (Shanghai Generay Biotech Ltd). The primers used for quantitative real-time PCR of viral NP RNA were 5′ –TGTGTATGGACCTGCCGTAGC – 3′ (sense) and 5′ – CCATCCACACCAGTTGACTCTTG – 3′ (antisense). The beta-actin was used as internal control of cellular RNAs, with primer sequences of 5′ –CGTGCGTGACATCAAGGAAGAAG – 3′ (sense) and reverse: 5′ –GGAACCGCTCGTTGCCAATG – 3′ (antisense).

Real-time PCR was performed using 100 ng of RNA and one-step qPCR kit (TOYOBO). Cycling conditions for real-time PCR were as follows: 90°C for 30 s, 61°C for 20 min, and 95°C for 1 min, followed by 45 cycles of 95°C for 15 s, 55°C for 15 s and 74°C for 45 s. As the loading control, we measured the level of beta-actin mRNA. Real-time PCR was conducted using the ABI Prism 7300 sequence detection system, and the data were analyzed using ABI Prism 7300 SDS software (Applied Biosystems).

### Immunization protocol

Six week-old female C57BL/6 mice(Beijing HFK Bioscience Ltd)were used in all mouse experiments. The feed is commercially available in the form of pellets and the diet is free of potentially pathogenic organisms. Water intake is about 15 ml/100 g/day. 50 C57/B6 mice were randomly divided into 5 groups. All mice were lightly anesthetized with 10 µl (6 mg/ml) Pentobarbital sodium/g mouse, followed by intranasal (i.n.) administration of wt, mutation A/PR8(1×10^4^ pfu), inactivated A/PR8 or PBS in 30 µl. Mice were monitored every day, including body weight, body temperature, activity and respiration rate. Mice were anesthetized by pentobarbital sodium with a dose of 60 µg/g and sacrificed by dislocation as their conditions deteriorated.

### Evaluation of virus replication in the respiratory tract of mice

To evaluate the level of replication of A/PR8 mutants in the respiratory tract of mice, on day 3, and 6 following inoculation, mice were anaesthetised by pentobarbital sodium with a dose of 60 µg/g and sacrificed by dislocation. Lungs were collected from 5 mice in each group, weighed, and homogenized in PBS to make 10% (wt/vol) (lungs) tissue homogenates. Tissue homogenates were clarified by centrifugation and viruses were titrated in 96-well tissue culture plates containing MDCK cells monolayer.

### Isolation of murine splenocytes

On day 6 following inoculation, after lungs were collected, spleens were harvested and mashed through the cell strainer into the petri dish and the cell strainer was washed with 5 mL RPMI-1640, and discarded. The suspended cells were transfered to a 15 ml tube and spun cells at 800×g for 3 minutes. The pellet was resuspended in 4 ml RBC lysis buffer and incubated for 4 minutes at RT. Finally the cells were washed with 10 mL RPMI-1640 and counted.

### Analysis of virus-specific CTL by tetramer-staining

Mouse CD8^+^-FITC and H-2D^b^-PE tetramer with the NP_366–374_ epitope ASNENMETM (Epigen Biotech, Beijing) were used. Single cell splenocyte suspensions were obtained and red blood cells were removed using erythrocyte lysis buffer. The splenocytes were washed 3 times with PBS, and 1×10^5^ cells were incubated with CD8^+^-FITC and H-2D^b^-PE tetramer on ice for 30 minutes. After incubation, the cells were washed twice with PBS. The labeled cells were analyzed by flow cytometry with a Guava Easycyte (Guava Technologies, Hayward, CA, USA). All FACS data were analyzed using FlowJo software (Tree Star, Ashland, OR, USA).

### Enzyme-linked immunospot (ELISPOT) for IFN-γ

Elispot assay was carried out according to instruction of Mouse IFN-γ pre-coated Elisport kit (Dakewe Biotech Company). Pre-coated plate was activated with 200 µl per well AM for 5–10 minutes at RT. PBMC from immunized animals were diluted to the required concentration (2×10^3^ cells per µl) and 100 µl cell suspension was added to wells and cultured for 16–20 hours at 37°C in CO_2_ incubator. After incubation and the supernatant was removed, 200 µl of iced water was added into each well to lyse cells for 10 minutes. The plate was washed with 200 µl of washing buffer for 1 minute, the wash was repeated 5–7 times. The conjugated secondary antibodies (Biotinylated antibody, dilute in dilution buffer) was added and incubated for 1 hour at room temperature. After washing streptavidin-HRP (dilute in dilution buffer) was added and incubated for 1 h at room temperature. The plate was washed again. The color was developed by adding enzyme substrate for 15 minutes at room temperature. To stop color development, the plate was rinsed in distilled water and dried. The spots were counted by an elispot reader (Cellular technology Ltd).

### Hemagglutination inhibition (HAI) assay

The HAI assay was used to determine the levels of H1N1-specific antibodies against homologous and heterologous viruses in mice sera post-infection. Prior to serological analysis, mice sera were treated with receptor-destroying enzyme (Sigma). Serum (0.1 ml) was mixed with 0.15 ml receptor-destroying enzyme, incubated at 37°C for 18 hours, and adjusted to a final 1:4 dilution by adding 0.15 ml of 0.9% sodium citrate, followed by incubation at 56°C for 45 minutes. Strain-specific serum HAI antibody titers were determined using 0.5% turkey RBCs (tRBCs), and the HAI titers were presented as the reciprocal value of the highest serum dilution that inhibited hemagglutination.

### Protection of influenza virus challenge in mice immunized by A/PR8 mutants

To determine efficacy of immunization by A/PR8 mutants against the challenge of A/PR8 in mice, we administered 30 µl of A/PR8 mutants (1×10^4^ pfu) intranasally to mice and challenged them with a lethal dose (1×10^7^ pfu) of A/PR8 one month after the final dose of vaccine. Control groups were immunized with PBS or A/PR8. On day 3 after challenge, lungs of mice were collected and virus titers were determined in MDCK cells as described above. Mice were anaesthetised by pentobarbital sodium with a dose of 60 µg/g and sacrificed by dislocation as their conditions deteriorated.
